# Pioneering Endoscopic Calcium-Electroporation in Gastric Cancer: A Case Series of an Emerging Therapeutic Approach

**DOI:** 10.3390/diseases13100340

**Published:** 2025-10-15

**Authors:** Giuliano Francesco Bonura, Noemi Gualandi, Paola Soriani, Pablo Cortegoso Valdivia, Tommaso Gabbani, Valentina Zadro, Federica Indulti, Gabriella Frassanito, Germana de Nucci, Mauro Manno

**Affiliations:** 1 Gastroenterology and Digestive Endoscopy Unit, Azienda USL di Modena, 41121 Modena, Italy; g.bonura@ausl.mo.it (G.F.B.); n.gualandi@ausl.mo.it (N.G.); p.soriani@ausl.mo.it (P.S.); t.gabbani@ausl.mo.it (T.G.); v.zadro@ausl.mo.it (V.Z.); f.indulti@ausl.mo.it (F.I.); g.frassanito@ausl.mo.it (G.F.); 2 Gastroenterology and Endoscopy Unit, University Hospital of Parma, University of Parma, 43126 Parma, Italy; cortegosopablo@yahoo.it; 3 Gastroenterology and Endoscopy Unit, ASST Rhodense, Garbagnate Milanese, 20024 Milan, Italy; germanadenucci1@gmail.com

**Keywords:** calcium electroporation, gastric cancer, upper gastrointestinal bleeding, iron-deficiency anemia, gastrointestinal endoscopy

## Abstract

Background/Objectives: Gastric cancer often presents at advanced stages with complications such as iron-deficiency anemia (IDA) due to chronic bleeding, representing a significant global health burden. Palliative management of bleeding tumors in frail patients remains challenging. This study evaluates the feasibility, safety, and efficacy of endoscopic calcium-electroporation (Ca-EP), a novel non-thermal ablation technique, for controlling bleeding in end-stage gastric cancer. Methods: Retrospective case series including consecutive patients with end-stage, bleeding gastric cancer and IDA requiring transfusions. Ca-EP was performed using the EndoVE system, which delivers bipolar electrical pulses (250 kHz) to induce reversible electroporation, enabling calcium influx and tumor cell apoptosis. Primary endpoints were clinical success (hemoglobin stabilization/reduced transfusions) and safety. Secondary endpoints included tumor regression, procedural time, and hospital stay. Results: Five patients (median age 81 years) were included. Clinical success was achieved in 80% (4/5) of patients, with reduced transfusion needs and stable hemoglobin levels. One patient required adjunctive hemostatic radiotherapy. No major or minor adverse events were reported, and all patients were discharged within 24 h. Procedural median time was 38 min (range: 22–65). Endoscopic follow-up in three patients showed mild tumor regression or stability. Three patients required repeat Ca-EP sessions due to recurrent bleeding. Conclusions: Endoscopic Ca-EP is a safe, minimally invasive palliative option for bleeding gastric cancer, offering sustained hemostasis and potential antitumor effects without systemic toxicity. Its feasibility in frail patients underscores its clinical relevance, though larger prospective studies are needed to optimize parameters and validate long-term outcomes.

## 1. Introduction

Gastric cancer is a major health concern, ranking as the fifth-most diagnosed cancer and the fifth leading cause of cancer-related death worldwide [1,2]. Early diagnosis is crucial to optimizing therapy. However, gastric cancer is frequently asymptomatic during the early phase, and most cases are diagnosed at an advanced stage and are metastatic or unfit for surgery, which contributes significantly to the high mortality rate [3]. At the time of diagnosis, weight loss, persistent abdominal pain and occult gastrointestinal bleeding, accompanied or not by iron deficiency anemia (IDA), are the most common symptoms [4].

IDA is a frequent complication in patients with gastric cancer, with a substantial impact on quality of life (QoL). Management often requires repeated blood transfusions and frequent hospitalizations, leading to increased healthcare costs [5,6]. In such cases, palliative and supportive therapies play a crucial role in the management of patients due to their frailty, and conventional oncological or surgical therapies may be poorly tolerated or even contraindicated because of perioperative risks including mortality and negative impact on QoL [7,8].

The EndoVE system, specifically designed to deliver calcium electroporation (Ca-EP), is an innovative palliative treatment that involves delivering electrical pulses directly to cells, increasing the permeability of the cell membrane and allowing calcium to enter tumor cells [9]. This influx of calcium induces cell death through apoptosis [10]. Ca-EP is a promising alternative to electrochemotherapy, which uses chemotherapeutic drugs that are more expensive and may cause general adverse events. This technique induces biochemical vulnerability of cancer cells and a local immune response that contributes to tumor ablation [11].

Ca-EP has demonstrated promising results in the treatment of patients with soft tissue sarcoma, cutaneous and pancreatic cancers, head and neck tumors [12,13]. Moreover, Ca-EP may determine the regression of cutaneous metastasis, without adverse events [14].

Recently, its utility has been evaluated in the palliative field of advanced symptomatic esophageal and colorectal cancer [15,16,17,18,19]. Endoscopic adaptation of Ca-EP offers a valuable relief and target control of obstructive, painful symptoms and bleeding events, limiting the morbidity and mortality associated with more invasive approaches [11].

Nevertheless, its use in gastric cancer has not been reported to date. The aim of this study is to evaluate the feasibility of Ca-EP in patients with bleeding gastric cancers.

## 2. Materials and Methods

This is a retrospective observational case series including all consecutive patients who underwent Ca-EP for bleeding gastric cancer at a single tertiary referral center.

Patients diagnosed with gastric cancer and IDA requiring blood transfusions were assessed for eligibility. Evaluation was conducted by a multidisciplinary team, which determined that no further standard treatment options were available and deemed the patients suitable candidates for Ca-EP therapy. Radiotherapy was also evaluated during multidisciplinary discussion. However, it carries-out potential adverse events and could be poor tolerated by the patient. Therefore, given the easier availability of the procedure, we decided to use Ca-EP as the first-line treatment, reserving hemostatic radiotherapy as a second-line option Patients who declined standard treatments were also considered eligible for Ca-EP.

Inclusion criteria were patient age ≥18 years, American Society of Anesthesiology score I-IV and ability to give informed consent. Exclusion criteria were patient age < 18 years, American Society of Anesthesiology score V, pregnancy or breastfeeding.

The study was conducted in accordance with the declaration of Helsinki.

All patients gave specific written informed consent for the procedure. The study was approved by the Research & Development office of our hospital.

### 2.1. Endpoints

The primary endpoint was to evaluate the feasibility of Ca-EP by assessing clinical success and safety.

Clinical success was defined as either stabilization of hemoglobin (Hb) levels or significant reduction in the required red blood cell (RBC) transfusions.

Safety was defined as the absence of major procedure-related adverse events, i.e., intraprocedural or delayed gastrointestinal perforation or bleeding.

Secondary endpoints were tumor regression evaluated by endoscopic assessment, minor adverse events (e.g., abdominal pain, fever, nausea, vomiting, etc.), procedural time and length of hospital stay.

### 2.2. Ca-EP Technique

Ca-EP is a technique that takes advantage of a physical process in which electric fields are applied directly to cell membranes to temporarily increase their permeability. This effect is not dependent on the type or histology of the cells, making it broadly applicable across different tissues. The process is governed by two key electrical parameters: the intensity of the electric field and the duration of the pulses. By adjusting these two variables, it is possible to induce either reversible or irreversible electroporation. In reversible electroporation, the electric pulses temporarily open pores in the cell membrane, allowing substances that normally cannot enter the cell—such as drugs, ions, or large molecules—to be introduced into the intracellular space. The membrane then gradually reseals, and the cell can survive the process. On the other hand, when very strong electric fields are applied for longer durations, the damage to the membrane becomes irreversible. In this case, the cell loses its structural integrity and cannot recover, ultimately leading to cell death. This distinction forms the basis for different therapeutic approaches, depending on whether the goal is to deliver agents into cells or to ablate the targeted tissue entirely [11,12].

All patients underwent a preliminary esophagogastroduodenoscopy (EGD) to assess the lesion targeted for treatment and to evaluate the feasibility of accessing it with the device.

Subsequently, the patient was hospitalized, and a Ca-EP procedure was performed.

All procedures were conducted under deep sedation with anesthetist-directed care, using propofol ± fentanyl and midazolam. Adverse events potentially related to sedation were also documented.

**Figure 1:**
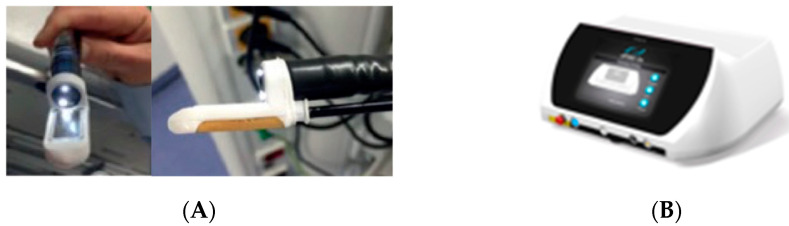
EndoVe (**A**) and ePore generator (**B**).

The EndoVE device is engineered for intraluminal gastrointestinal procedures and is used in conjunction with a standard endoscope (Figure 1A). It features an expandable ring designed to securely accommodate and integrate with the endoscope. Once mounted over the scope, the EndoVE chamber facilitates precise positioning at the treatment site.

Within the chamber are two gold-plated conductive electrodes, which are connected to a vacuum-assisted aspirator. This setup enables the target tissue to be drawn into the chamber and immobilized via suction, ensuring stable and localized contact with the electrodes.

Electroporation is delivered through these electrodes using the ePORE generator, a next-generation device developed by Mirai Medical (Figure 1B). The ePORE system delivers bipolar electrical pulses at 250 kHz, a frequency selected to minimize muscle contractions and procedural pain. This short-duration electric field induces reversible electroporation in the aspirated tissue, allowing for targeted therapy with reduced patient discomfort.

### 2.3. Follow-Up

All patients were observed for 24 h and discharged the day after the procedure if asymptomatic. One-week after the procedure, a full blood count was repeated, and a clinical evaluation was performed. If the Hb level had decreased by more than 2 g/dL, or if signs of gastrointestinal bleeding (e.g., melena) were observed during the clinical evaluation, a second Ca-EP procedure was scheduled three-weeks after the initial treatment. Conversely, if the Hb level remained stable or increased, clinical (through outpatient visit) and biochemical follow-up (including full blood count) continued with monthly evaluations. In cases of recurrent gastrointestinal bleeding or recurrent anemia, a second Ca-EP procedure was performed. During follow-up, we also evaluated any adverse events related to the Ca-EP procedures.

### 2.4. Data Collection and Analysis

Data included demographic, procedural, and outcome variables extracted from electronic medical records. Given the small cohort and exploratory nature of this study, descriptive statistics were primarily employed. Continuous variables were expressed as median (range) due to the non-normal distribution expected in a limited cohort, while categorical variables were reported as percentages. The Wilcoxon signed-rank test was used for the comparison of pre- and post-procedural Hb median values, as it is the most appropriate non-parametric test for paired samples of small size. Given the reduced statistical power inherent to the study’s design, a *p*-value < 0.05 was considered statistically significant. All analyses were performed using R software (version 4.5.1 for macOS; R Foundation for Statistical Computing, Vienna, Austria).

## 3. Results

Between February 2024 and May 2025, five (*n* = 5) patients with bleeding gastric cancer and IDA requiring blood transfusion underwent Ca-EP treatment. All patients were male, with a median age of 81 years (range, 72–86). The tumor locations varied: two lesions were in the fundus, two in the body, and one in the cardia. Three patients received two consecutive Ca-EP sessions, while the remaining two were treated with a single session. Baseline patients’ characteristics are resumed in Table 1.

Clinical success was achieved in four out of five patients (80%). One patient (20%) showed persistent IDA despite Ca-EP treatment, leading to the use of hemostatic radiotherapy as an additional intervention.

**Figure 2:**
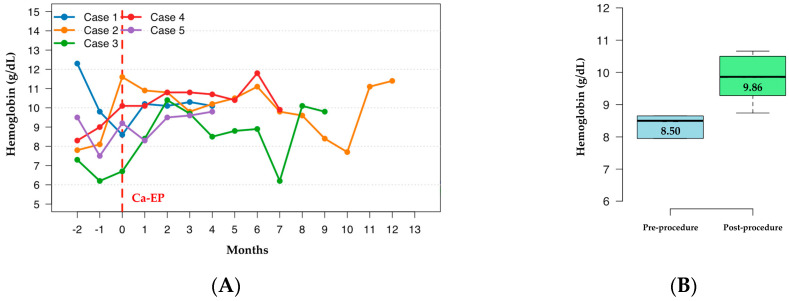
Hb levels during the observational period (**A**). Comparison of median Hb levels before and after Ca-EP treatment (**B**).

Hb dynamics are demonstrated in Figure 2A. For the purpose of statistical comparison and to ensure data completeness, the analysis was limited to the first 4 months post-procedure, as this was the timepoint with available Hb values for all five patients. Quantitative analysis showed an increase in median hemoglobin levels from 8.50 g/dL (range: 6.75–11.05) pre-procedurally to 9.86 g/dL (range: 8.74–10.66) during this initial post-procedural period (Figure 2B). Although this upward trend was observed in 4 out of 5 patients, the difference did not reach formal statistical significance (*p* = 0.178, Wilcoxon signed-rank test), likely due to the limited sample size reducing statistical power to detect smaller effect sizes.

The procedure was well tolerated. No major or minor adverse events were reported, and all patients were discharged the day after the procedure, asymptomatic. The median procedural time was 38 min (range, 22–65). During follow-up, three patients underwent endoscopic evaluation. In two of these cases, a mild regression of the tumor tissue was observed, while in the third case the lesion remained stable in size, with no evidence of disease progression.

### 3.1. Case 1

**Figure 3:**
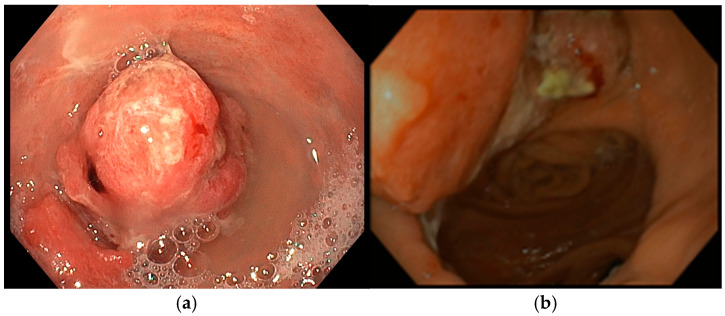
(**a**) Pre-procedure gastric cancer, (**b**) one month after EndoVe treatment.

A 72-year-old man with a medical history of cholecystectomy and anterior rectal resection for rectal adenocarcinoma with a definitive colostomy underwent partial gastrectomy for gastric adenocarcinoma in July 2022. In December 2023, he was admitted to our unit for an upper gastrointestinal bleeding (UGIB) associated with IDA, requiring two units of RBC transfusions. Urgent EGD revealed a 25 mm bleeding recurrent lesion in the remnant stomach (Figure 3a), located near the gastrojejunal anastomosis, which was initially managed with topical hemostatic agents. Subsequent radiologic follow-up identified liver metastases, and during multidisciplinary team discussion, palliative chemotherapy was recommended. However, the patient declined further chemotherapy or radiotherapy. Given the persistent bleeding and refusal of systemic therapy, the patient was considered eligible for Ca-EP. At the time of the first Ca-EP session (25 Pulses, 1011 Volts) in February 2024, his Hb level was 8.6 g/dL. One-week after the procedure, Hb had risen to 10.2 g/dL, indicating initial clinical benefit. However, two weeks later, the patient experienced a new episode of melena and a drop in Hb to 8.8 g/dL requiring one RBC transfusion, prompting a second Ca-EP session (25 Pulses, 998 Volts). Endoscopic evaluation demonstrated a slight reduction in tumor size with no active bleeding as shown in Figure 3b where a mild regression of the bulky component of the lesion was observed. Following the second treatment, the patient did not show bleeding recurrence or anemia (Hb levels 9.2–10.2 g/dL). Unfortunately, four months later, the patient died due to an ischemic stroke.

### 3.2. Case 2

**Figure 4:**
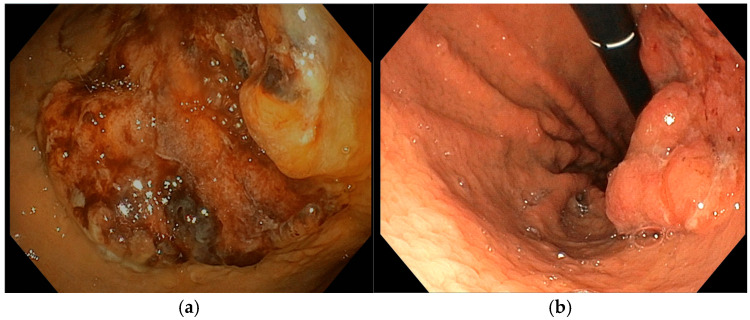
(**a**) Pre-procedure gastric cancer, (**b**) a few weeks after EndoVe treatment.

An 86-year-old male with significant comorbidities (type II diabetes mellitus, essential hypertension, dyslipidemia, chronic kidney disease, dilated hypokinetic cardiomyopathy, mild cognitive impairment and atrial fibrillation on direct oral anticoagulation) underwent EGD in March 2024 for IDA requiring three RBC transfusions (Hb 7.5 g/dL) that revealed a 35 mm actively bleeding gastric lesion (Figure 4a), positive for gastric adenocarcinoma at histopathological biopsy. Following a multidisciplinary discussion, curative or aggressive therapeutic options were deemed unfeasible due to the patient’s advanced age and multiple comorbidities. As a result, the patient was considered eligible for Ca-EP treatment. In June 2024, the patient underwent the first session of Ca-EP (38 Pulses, 991 Volts). Two months later, Hb levels decreased from 10.7 to 8.6 g/dL. A second session of Ca-EP was therefore performed (38 Pulses, 992 Volts). Endoscopic evaluation showed a slight reduction in the size of the gastric lesion with no evidence of spontaneous bleeding (Figure 4b). Following the second treatment, the patient experiences a single episode of recurrent anemia in February 2025 requiring two RBC transfusion. As of the latest follow-up, the patient remains alive, clinically stable, and had no further significant episodes of anemia.

### 3.3. Case 3

**Figure 5:**
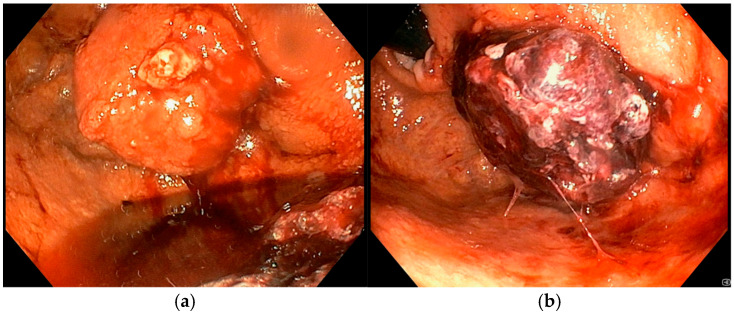
(**a**) Pre-procedure gastric cancer, (**b**) gastric cancer immediately after EndoVe treatment.

In May 2024, an 81-year-old man with no significant comorbidities was admitted to our department for evaluation and management of severe IDA (Hb 7.1 g/dL). An EGD revealed a bleeding 30 mm gastric tumor located in the fundus (Figure 5a). Cross-sectional imaging confirmed the presence of liver metastases, consistent with a diagnosis of stage IV gastric cancer. Following multidisciplinary discussion, the patient was deemed a suitable candidate for systemic chemotherapy, and treatment was initiated with trastuzumab and capecitabine, given the tumor’s HER2-positive status. Despite systemic therapy, the patient remained transfusion-dependent due to ongoing UGIB. To control the bleeding locally, two sessions of Ca-EP were performed. The first Ca-EP session was carried out in July 2024 (22 Pulses, 991 Volts), leading to a modest, short-term reduction in bleeding. Figure 5b shows the effect of Ca-EP on the lesion that produce an immediate hemostatic effect. However, the patient continued to require weekly blood transfusions, prompting a second Ca-EP session (22 Pulses, 995 Volts) approximately one month later. During the second procedure a topical hemostatic agent was used during endoscopy to control active bleeding. Despite these interventions, endoscopic therapy alone was insufficient to achieve durable hemostasis. Given the persistent and symptomatic bleeding, the need for weekly blood transfusion, the patient was referred to the radiation oncology department for hemostatic radiotherapy. A total dose of 30 Gy was administered, which led to successful bleeding control. In January 2025, the patient presented again with acute anemia (Hb 7.8 g/dL). EGD was repeated but did not identify any active bleeding sources. As of the most recent follow-up, the patient requires periodic RBC transfusions.

### 3.4. Case 4

**Figure 6:**
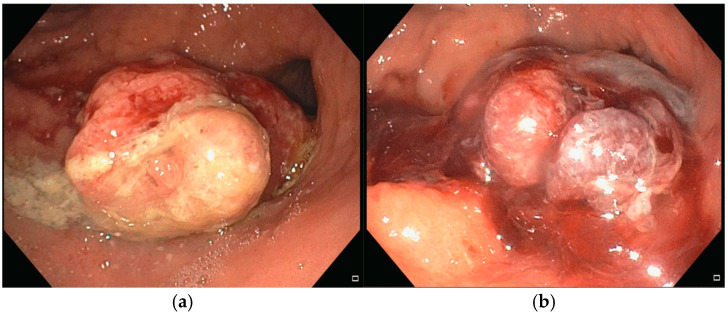
(**a**) Pre-procedure gastric cancer, (**b**) gastric cancer immediately after EndoVe treatment.

An 85-year-old man with Alzheimer’s dementia, diabetes mellitus, ischemic cardiomyopathy, and atrial fibrillation on direct per oral anticoagulants underwent a gastric wedge resection for T2N0M0 corpus bleeding adenocarcinoma in October 2023. In October 2024, he was hospitalized due to UGIB. Urgent EGD was performed showing a recurrent 30 mm sub-stenotic gastric lesion involving the body-antrum region (Figure 6a). After multidisciplinary discussion, he was considered unsuitable for further therapies other than palliative care. He underwent only one Ca-EP session in November 2024 (22 Pulses, 991 Volts) (Figure 6b). At the time of discharge Hb was 9.5 g/dL. During the subsequent follow-up, patient did not experience new episodes of UGIB, and Hb levels varied within 9.0 and 10.6 g/dL. He never required blood transfusions but only received intravenous iron supplementation before the procedure.

### 3.5. Case 5

**Figure 7:**
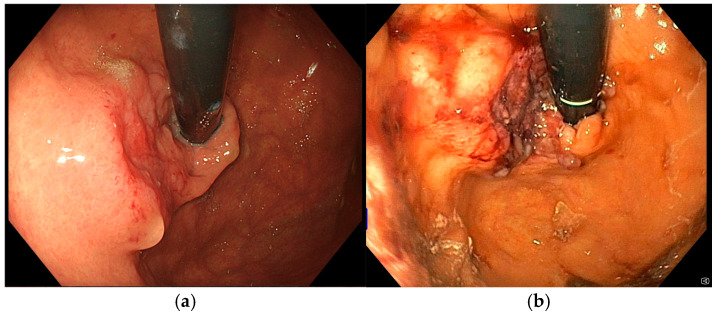
(**a**) Pre-procedure gastric cancer, (**b**) gastric cancer immediately after EndoVe treatment.

An 80-year-old male with a complex medical history significant for multiple comorbidities (essential hypertension, type II diabetes mellitus, vascular encephalopathy, prior left hemicolectomy for colorectal cancer, multiple treatments for colonic angiodysplasia, and a recent percutaneous transluminal angioplasty with stent placement in the popliteal artery due to peripheral arterial disease, after which he was started on dual antiplatelet therapy) presented with severe IDA (Hb 5.9 g/dL) in March 2025. No active bleeding or mucosal lesions were identified on colonoscopy, while EGD identified a 35 mm lesion at the cardia, easily bleeding upon contact with the endoscope (Figure 7a). Given the patient’s advanced age, frailty, and significant comorbidities, curative or aggressive oncological interventions were deemed unfeasible after multidisciplinary evaluation. Therefore, the patient was considered eligible for Ca-EP, which was carried on in April 2025 (22 pulses were applied, 1005 Volts) (Figure 7b). Follow-up evaluations revealed no signs of upper gastrointestinal bleeding or further decline in hemoglobin levels.

## 4. Discussion

To the best of our knowledge, this is the first case series reporting clinical application of endoscopic Ca-EP for palliative treatment of inoperable bleeding gastric cancer.

The procedure has demonstrated efficacy and safety, with promising preliminary results in terms of clinical benefit in this small cohort of elderly, fragile patients with limited therapeutic options [7,8].

IDA is a frequent complication of chronic gastrointestinal bleeding tumors and has a substantial impact on patients’ QoL, as repeated hospitalizations and transfusions are often required [5,6].

Our results showed that Ca-EP, a non-thermal tumor ablation technique, turned out to be a minimally invasive, and well-tolerated procedure that provided symptomatic relief, reducing bleeding-related morbidity.

Clinical success was achieved in 80% of patients. No major nor minor AEs were recorded, all patients were discharged within 24 h post-procedure. These findings are consistent with prior Ca-EP studies in colorectal and esophageal cancers, which reported minimal procedural risks and good tolerability [11,15,16,17,19]. Since this was our first experience with the use of Ca-EP, we chose to proceed with inpatient management. However, the procedure could have been safely performed on an outpatient basis with only brief post-procedural observation [20].

Three patients had recurrent cancer bleeding, requiring subsequent sessions of Ca-EP, highlighting the potential need for staged or maintenance treatments in this setting. In fact, prior studies [15,16,17,18] used multiple Ca-EP sessions to provide sustained palliative benefit in esophageal and colorectal bleeding cancers.

Importantly, none of our patients required emergency surgery or had procedure-related complications, reinforcing the safety profile of endoscopic Ca-EP.

In three patients, endoscopic follow-up revealed mild tumor regression or stability, suggesting potential antitumor activity. While this was not a primary endpoint, it aligns with previous reports of Ca-EP’s capacity to induce local tumor necrosis and immunogenic cell death via calcium-mediated apoptosis [9,10,12,14].

Although preliminary and based on a small patient cohort, these findings suggest a dual benefit—both hemostatic and potentially cytoreductive—warranting further investigation.

In contrast, one patient did not achieve adequate bleeding control with Ca-EP alone and required hemostatic radiotherapy. This reflects the variability of the tumor response, which may be related to differences in tumor vascularity, location, or electrical conductivity. Future studies could explore predictive markers of Ca-EP efficacy or optimize electroporation parameters (e.g., voltage, pulse duration) to enhance therapeutic outcomes [10,12].

Compared to electrochemotherapy, Ca-EP is a simpler, more accessible technique that avoids the use of chemotherapeutic agents, reducing systemic toxicity and cost [9,11]. This is particularly relevant in frail populations where chemotherapy may be contraindicated. Furthermore, the endoscopic delivery platform used in this study (EndoVE) allows for precise targeting of intraluminal lesions, expanding the scope of Ca-EP beyond cutaneous or superficial tumors [13,14].

This study has several limitations. First, it is a small observational series without a control group, limiting generalizability. Second, objective measures of tumor burden (e.g., volumetric imaging, histological response) were not routinely obtained; lesion size was assessed based on the experience of the endoscopist performing the procedure and by comparing pre- and post-procedure images. Third, long-term follow-up was limited, and survival outcomes were not assessed. Despite these limitations, this work provides foundational clinical evidence supporting the role of Ca-EP in gastric cancer palliation.

## 5. Conclusions

Ca-EP appears to be a safe and efficacy palliative option for managing inoperable bleeding gastric cancer in frail patients. It offers hemostatic control and possibly tumor regression without the burden of systemic toxicity, improving QoL. Further prospective studies with larger cohorts and longer follow-up, including also objective measures of tumor regression, are warranted to confirm these findings and refine patient selection criteria.

## Figures and Tables

**Table 1:** Baseline patients’ characteristics.

Patient n	Sex	Age at the Diagnosis	Tumor Location	Size (mm)	Previous Treatment	Treatment n	Date of I Treatment	Pulses n, Voltage	Duration (min)	Date of II Session	Pulses n, Volts	Duration (min)	Effective
1	M	72	body	25	Surgery + Ct	2	02/2024	25 1011 V	60	03/2024	25 998 V	24	Yes
2	M	86	body	35	None	2	06/2024	38 991 V	30	08/2024	38 992 V	35	Yes
3	M	81	fundus	30	Ct	2	07/2024	22 991 V	38	08/2024	22 995 V	55	No
4	M	85	Body	30	Surgery + Ct	1	11/2024	22 991 V	22	\	\	\	Yes
5	M	80	cardia	35	None	1	04/2025	22 1005 V	45	\	\	\	Yes

Ct, Chemotherapy; V, Volt.

## Data Availability

The original contributions presented in this study are included in the article. Further inquiries can be directed to the corresponding author(s).

## Abbreviations

The following abbreviations are used in this manuscript:

Ca-EP	(calcium-electroporation)
EGD	(esophagogastroduodenoscopy)
Hb	(Hemoglobin)
IDA	(iron deficiency anemia)
QoL	(quality of life)
RBC	(red blood cell)
UGIB	(upper gastrointestinal bleeding)
